# Stratification of disease progression in a broad spectrum of degenerative cerebellar ataxias with a clustering method using MRI-based atrophy rates of brain structures

**DOI:** 10.1186/s40673-017-0068-4

**Published:** 2017-06-29

**Authors:** Rie Sasaki, Futaba Maki, Daisuke Hara, Shigeaki Tanaka, Yasuhiro Hasegawa

**Affiliations:** Department of Internal Medicine, Division of Neurology, St. Marianna University School of Medicine, 2-16-1 Sugao, Miyamae, Kawasaki, Kanagawa 216-8511 Japan

**Keywords:** MRI, Cerebellar volume, Spinocerebellar degeneration, Multiple system atrophy, Corpus callosum, Spinocerebellar ataxia, Cluster analysis

## Abstract

**Background:**

The rate of disease progression differs among patients with degenerative cerebellar ataxia. The uncertain natural course in individual patients hinders clinical trials of promising treatments. In this study, we analyzed atrophy changes in brain structures with cluster analysis to find sub-groups of patients with homogenous symptom progression in a broad spectrum of degenerative cerebellar ataxias.

**Methods:**

We examined 48 patients including 21 cases of spinocerebellar ataxia (SCA), 17 cases of the cerebellar type of multiple system atrophy (MSA-C), and 10 cases of cortical cerebellar ataxia (CCA). In all patients, at least two sets of evaluations including magnetic resonance imaging (MRI) and the International Cooperative Ataxia Rating Scale (ICARS) scoring were performed. The median number (min-max) of follow-up studies in each patient was three (2–6), and the mean follow-up period was 3.1 ± 1.6 years. The area of the corpus callosum on midsagittal images and the cerebellar volume were measured using MRI, and these values were divided by the cranial antero-posterior diameter of each patient to correct for individual head size differences as an area index (Adx) and a volume index (Vdx), respectively. The annual changes in Adx, Vdx, and ICARS score were calculated in each patient, and atrophy patterns in patients were categorized with cluster analysis.

**Results:**

The annual atrophy rates for the corpus callosum (Adx) and cerebellum (Vdx) and symptom progression differed significantly by subtype of cerebellar ataxia (*p* = 0.026, 0.019, and 0.021, respectively). However, neither the annual atrophy rate of Adx nor Vdx was significantly correlated with the annual increase in the ICARS score. When the patients were categorized into three clusters based on the annual changes in Adx and Vdx, the annual increase in the ICARS score was significantly different among clusters (2.9 ± 1.7/year in Cluster 1, 4.8 ± 3.2/year in Cluster 2, and 8.7 ± 6.1/year in Cluster 3; *p* = 0.014).

**Conclusions:**

The annual increase in the ICARS score can be stratified by cluster analysis based on the atrophy rates of the corpus callosum and cerebellum. Further studies are warranted to explore whether these simple MRI methods could be used for random allocation of a broad spectrum of patients with degenerative cerebellar ataxia in clinical trials.

## Background

Degenerative cerebellar ataxia comprises heterogeneous diseases that mainly affect the cerebellum with various degrees of pathological changes in other brain structures. Many types of neurodegenerative ataxia are inherited as autosomal dominant traits and have been named spinocerebellar ataxia (SCA) [1]. The current molecular classification of SCAs corresponds to the order in which the responsible genes were described, and more than 30 types of SCAs have been identified [2–5]. Genetic testing is available for several types of SCAs in routine clinical practice, but 10–20% of cases of dominant SCA are due to unknown mutations [6]. Moreover, the rate of progression may vary, even in patients with the same genotype [7]. Among subtypes of degenerative cerebellar ataxia, the sporadic form is the most common form in Japan (67.2%) and includes cortical cerebellar ataxia (CCA) [7, 8] and the cerebellar type of multiple system atrophy (MSA-C) [9]. A nationwide registry system of ‘intractable diseases’ in Japan demonstrated the prevalence of CCA in Japan has reached about 9000 [6, 7]. CCA is nearly synonymous with sporadic adult-onset ataxia of unknown origin [10] and idiopathic cerebellar ataxia [11] in Western countries. The concept of CCA as a disease is somewhat ambiguous, because the diagnosis is made by ruling out acquired and genetic causes of ataxia, as well as MSA. CCA may not be necessarily sporadic, and the differential diagnosis from MSA-C in the early stages is difficult [8].CCA may be a mixed disease entity with recessive inheritance or dominant inheritance with very low penetrance [8]. Differential diagnosis of degenerative cerebellar ataxia in the early stage remains challenging.

Since Sobue et al. reported that intravenous administration of taltirelin hydrate, a synthetic thyrotropin-releasing hormone analogue, improves ataxia in these diseases in 1983 [12], taltirelin hydrate has been the most widely used drug in Japan for the treatment of neurodegenerative ataxia. Although several drugs have been tested in randomized controlled trials, including lithium [13] and varenicline [14] in patients with SCA3 and riluzole [15] in patients with a broad spectrum of neurodegenerative ataxias, no new treatments have been approved in over 40 years. The uncertainty of the natural course in individual patients confounds determination of the most appropriate study design to verify the efficacy of promising drugs [16–18].

Cerebellar volume measurement using magnetic resonance imaging (MRI) could be used as an imaging biomarker to predict progression rates in individual patients with these diseases [1, 19–21]. We demonstrated that the cerebellar volume obtained from MRI correlates well with the International Cooperative Ataxia Rating Scale (ICARS) score in a broad spectrum of degenerative cerebellar ataxias [22]. We also confirmed that the annual atrophy rate of the cerebellar volume and the annual progression of the ICARS score were significantly different among subtypes of cerebellar degeneration [23]. However, categorizing individual symptom progression only by the atrophy rate of the cerebellum is not feasible. Atrophy of the corpus callosum, the quantitative evaluation of which is easy on midsagittal MR images, has been used as an indirect but sensitive index of cortical neuronal loss [24, 25]. Cluster analysis has been used to identify phenotypes that exhibit differences in clinical response to treatment algorithms [26–29]. The clustering method is a multivariate statistical procedure used to create homogenous groups of subjects as suggested by the data, but not defined prior to analysis. In this study, we investigated whether categorization by cluster analysis using atrophy rates of the corpus callosum and cerebellum could be used as an imaging biomarker to predict gross neurological deterioration as evaluated by ICARS.

## Methods

### Patients

We retrospectively analyzed a database of patients with degenerative cerebellar ataxia who were treated in our hospital from January 2004 to April 2013. A total of 111 patients were followed using a standardized follow-up protocol including MRI and neurological examination with ICARS. To determine the annual atrophy rates of the cerebellum and corpus callosum, we chose 48 patients (24 males; mean age 60.5 ± 11.3 years) from our data set using the following inclusion criteria: 1) at least two sets of evaluations including MRI and ICARS scoring were performed, 2) at least one follow-up study was performed after 18 months (allowance, ±6 months), and 3) ICARS scoring was performed on the same day of MRI examination (allowance, ±45 days).

Diagnosis of MSA-C was made in accordance with the second consensus statement including MRI findings [9]. Patients with a family history suggestive of dominant inheritance were diagnosed with SCA. After obtaining informed consent for genetic testing, patients were screened for SCA1, SCA2, SCA3, and SCA6. Further screening for SCA7, SCA8, SCA17, and SCA31 was performed in patients in whom the first screening was negative. When the other rare SCAs were suggested, further studies were performed according to the flow chart suggested by the Study Group on Ataxic Diseases and supported by the Ministry of Health, Labour and Welfare of Japan [6]. Patients with autosomal recessive cerebellar ataxia, such as Friedreich ataxia, ataxia-telangiectasia, ataxia with vitamin E deficiency, etc. were excluded. The diagnosis of CCA was made based on the following criteria: progressive ataxia; disease onset after 20 years of age; no acute or subacute disease onset; informative and negative family history or no evidence of a causative gene mutation, at least the negative results for screening test of SCA1, SCA2, SCA3 and SCA6; no established symptomatic cause; and no possible or probable MSA [8]. For the subtype categorization of patients, we adopted their final diagnosis.

This study was conducted in a single hospital. The study protocol was approved by the St. Marianna University Bioethics Committee, and written informed consent was obtained from normal volunteers. Written informed consent from patients was waived because of the retrospective analysis of anonymized data.

### Measurements of brain structures

**Fig. 1:**
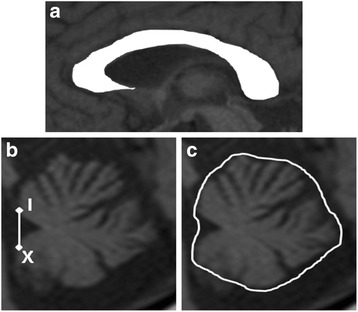
Segmentation of the corpus callosum and the cerebellum on MR images**.** The corpus callosum on a midsagittal T1-weighted image was extracted with the automated segmentation tool (**a**). The total area of the corpus callosum (mm^2^) was divided by the individual cranial AP diameter to correct for the individual head size differences as an area index (Adx). The cerebellum was defined as the area lateral to the line connecting the anterior lobe of the cerebellum (I segment) and cerebellar flocculus (X segment) (**b**). On the central slice, the fourth ventricle and surrounding cerebellar tissue were selected, and the cerebellum was automatically extracted using the imaging analyzer (**c**)

**Fig. 2:**
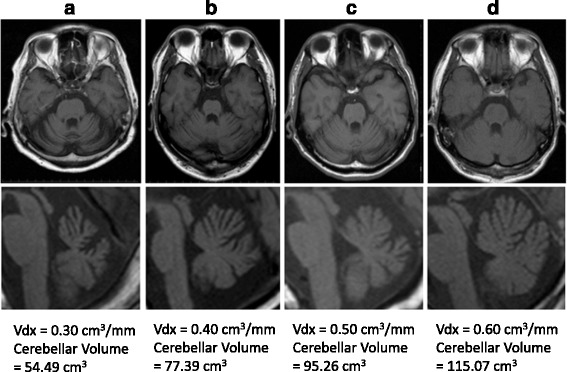
Representative images of cerebellar atrophy (T1-weighted image) and Vdx (**a**-**d**). Vdx is the cerebellar volume index (Vdx = cerebellar volume/cranial AP diameter). The mean Vdx value in normal adults (mean age, 64.2 ± 18.7 years; cranial AP diameter, 18.2 ± 1.1 cm) is 0.65 ± 0.06 cm^3^/mm. Vdx can be used to quantify the decrease in volume due to cerebellar atrophy, which is difficult to evaluate with visual qualitative assessment alone

Brain MRI was performed in all patients using a 1.5-T scanner (EXCELART®, Toshiba Medical Systems Co., Ltd., Tokyo, Japan; Achieva Nova-Dual®, Philips Electronics, Tokyo, Japan). Morphometric analyses of brain structures were performed with an image analyzer (TRI/3D–VOL; Ratoc System Engineering, Tokyo, Japan) using Digital Imaging and Communications in Medicine data from T1-weighted sagittal images (repetition time, 520 ms; echo time, 15 ms; repetition time, 520 ms; slice thickness, 4 mm; matrix, 272 × 256 sagittal sections). The corpus callosum on midsagittal images was extracted using the automated segmentation tool in the imaging analyzer (Fig. 1a), and we measured the total area (mm^2^). Cerebellar volume was measured as described elsewhere [22, 23]. Briefly, the cerebellum was defined as the area lateral to the line connecting the anterior lobe of the cerebellum and cerebellar flocculus. On the central slice, the fourth ventricle and surrounding cerebellar tissue were selected, and the cerebellum was automatically extracted using the imaging analyzer (Fig. 1b, c). The ventricle around the cerebellum, with a pixel number different than the cerebellar tissue, was separated from the cerebellum. Cerebellar volume was determined by linear interpolation from the automatically extracted cerebellar tissue area and slice thickness. In all patients, cranial antero-posterior (AP) diameter, which was defined as the distance between two points at which the skull and the anterior commissure – posterior commissure (AC-PC) line intersected, was measured. For statistical analysis, the area of the corpus callosum and the volume of the cerebellum of all patients were divided by each cranial AP diameter to correct for the individual head size differences as an area index (Adx) and a volume index (Vdx), respectively. Representative images of the cerebellum are shown in Fig. 2.

The normal values for Adx and Vdx in 30 healthy adults (mean age, 64.2 ± 18.7 years, cranial AP diameter, 18.2 ± 1.1 cm, body height, 163.5 ± 6.7 cm) were 3.80 ± 0.50 mm^2^/mm and 0.65 ± 0.06 cm^3^/mm, respectively. Inter-rater variability and test-retest reliability for the MRI-based morphometry were calculated using 12 randomly selected patients from our data set. Adx and Vdx were measured three times by three experienced neurologists who were blinded to the patient’s clinical information. The intraclass correlation coefficients for Vdx were 0.988 for inter-rater variability and 0.994 for test-retest reliability. The annual atrophy volume and the annual progression as measured with the ICARS score were calculated using a linear regression model in each patient.

### Statistical analyses

Hierarchical cluster analysis was used to define three homogeneous groups of individuals based on the annual atrophy changes in the corpus callosum (Adx) and cerebellum (Vdx) irrespective of etiology. Ward’s minimum-variance clustering method was used to create the best set of clusters for each possible number of clusters. Hierarchical clustering methods were first used to assign each individual to his or her own cluster. Then, the most similar pairs of clusters (in terms of the chosen distance metric) were merged into a new cluster, resulting in one less cluster.

Data are presented as the means and standard deviation (SD), unless otherwise indicated. In each patient, Adx and Vdx at the last follow-up visit were subtracted from the initial values, and these subtracted values were divided by the follow-up periods between the first and the last evaluation. Thus, the annual atrophy area in Adx and the annual atrophy volume in Vdx were calculated for each patient. The annual progression in the ICARS score was calculated by using the initial and last ICARS scores. Comparisons among three subgroups were made using ANOVA and the post-hoc Dunnett’s test. Values of *p* < 0.05 were considered significant. All statistical analyses were performed using SPSS version 22 (IBM SPSS Statistics for Windows; IBM Corp, Armonk, NY).

## Results

**Fig. 3:**
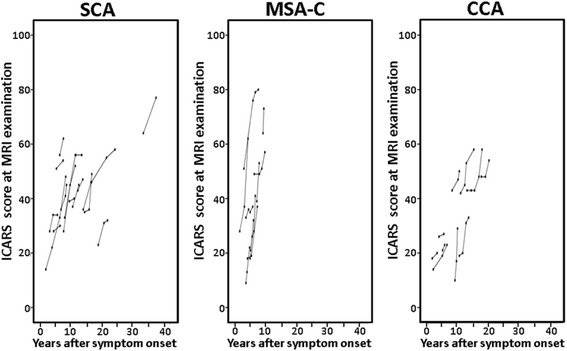
Serial changes in the ICARS score of individual patients by subtype of degenerative cerebellar ataxia. The median number (min-max) of follow-up studies was three (2–6). The mean follow-up period (min-max) was 3.1 ± 1.6 (1.25–7.58) years

We examined 48 patients including 21 patients with SCA (six with SCA6, four with SCA3, three with SCA2, three with SCA1, one with SCA31, and four for whom the type of SCA was unknown), 17 patients with MSA-C, and 10 patients with CCA. Patient characteristics and annual changes in Adx, Vdx, and the ICARS score are shown in Table 1. A total of 169 follow-up studies were performed in these patients. The median number (min-max) of follow-up studies was three (2–6). The mean follow-up period (min-max) was 3.1 ± 1.6 (1.25–7.58) years. The mean age at symptom onset and the age at entry into the study were younger for SCA than the other types (*p* = 0.005 and 0.046, respectively). The mean interval between onset and study entry for MSA-C was the shortest (*p* = 0.004). We found no significant difference in the ICARS score at the first evaluation among patients with SCA, MSA-C, and CCA. Both the annual atrophy of the corpus callosum area (Adx) and the cerebellar volume (Vdx) were highest in MSA-C (*p* = 0.026 and 0.019, respectively). The annual increase in the ICARS score was also highest in MSA-C (9.8 ± 6.1 points/year, *p* = 0.021). Serial changes in the ICARS score of individual patients were plotted against years after symptom onset in Fig. 3 **.** Neither the annual atrophy rate of Adx nor Vdx was significantly correlated with the annual increase in the ICARS score.

**Table 1:** Patient characteristics and annual atrophy changes in the corpus callosum and cerebellum

	Total (*n* = 48)	SCA (*n* = 21)	MSA-C (*n* = 17)	CCA (*n* = 10)	*p* value
Age at onset (years)	52.6 ± 12.5	46.2 ± 13.1*	57.5 ± 10.0	57.6 ± 9.2	0.005
Age at study entry (years)	60.5 ± 11.3	56.6 ± 12.5*	61.4 ± 9.4	67.1 ± 9.0	0.046
Male (%)	50%	62%	47%	30%	0.241
Body height (cm)	159.3 ± 9.0	160.3 ± 9.2	163.0 ± 8.0	154.7 ± 8.2	0.124
AP diameter of the cranium (mm)	181.4 ± 8.6	180.6 ± 8.4	185.0 ± 6.3	177.2 ± 10.7	0.061
Follow-up period (years)	3.1 ± 1.6	3.1 ± 1.7	2.7 ± 1.1	3.6 ± 1.8	0.522
Follow-up period (min-max)	(1.25–7.58)	(1.50–7.58)	(1.25–4.67)	(1.25–6.00)	
Number of follow-up studies, median (min-max)	3 (2–6)	3 (2–6)	3 (2–6)	4 (2–6)	0.083
Interval between onset and the first evaluation (years)	8.0 ± 6.4	10.4 ± 7.3	4.0 ± 3.3*	9.4 ± 5.3	0.004
Interval between the first and last evaluation (years)	2.6 ± 1.5	2.6 ± 1.6	2.2 ± 1.3	3.1 ± 1.6	0.308
ICARS score at the first visit	35.5 ± 16.5	36.7 ± 12.9	35.8 ± 23.0	33.2 ± 14.0	0.871
Initial corpus callosum area (mm^2^)	598.2 ± 79.2	575.2 ± 80.6	657.9 ± 84.9	587.0 ± 47.8	0.042
Initial corpus callosum area index (Adx)	3.38 ± 0.45	3.33 ± 0.50	3.46 ± 0.47	3.32 ± 0.26	0.622
Annual atrophy area in Adx	−0.11 ± 0.08	−0.09 ± 0.07	−0.15 ± 0.10	−0.08 ± 0.05	0.026
Initial cerebellar volume (cm^3^)	84.9 ± 13.7	88.1 ± 12.7	85.5 ± 8.8	78.4 ± 17.9	0.276
Initial cerebellar volume index (Vdx)	0.47 ± 0.09	0.48 ± 0.08	0.45 ± 0.09	0.47 ± 0.11	0.725
Annual atrophy volume in Vdx	−0.04 ± 0.02	−0.02 ± 0.02	−0.04 ± 0.02^†^	−0.01 ± 0.03	0.019
Annual increase in ICARS score	4.4 ± 4.5	3.5 ± 2.9	9.8 ± 6.1*	2.9 ± 2.8	0.021

*p < 0.05 vs. the other two subtypes (post-hoc analysis), ^†^ *p* < 0.05 vs. CCA (post-hoc analysis)

**Fig. 4:**
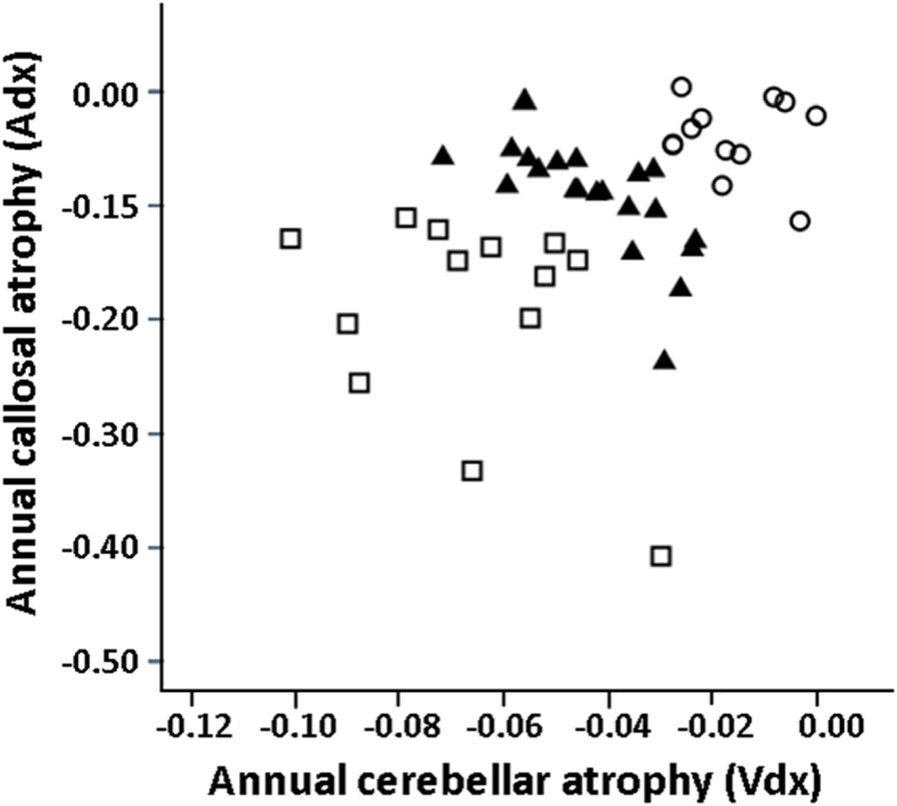
Categorization of patients by cluster analysis. Based on the annual atrophy rates of corpus callosum (Adx) and cerebellum (Vdx), patients were classified into three clusters (○: cluster 1, ▲: cluster 2, □: cluster 3)

The annual callosal (Adx) atrophy was plotted against the annual cerebellar volume (Vdx) atrophy as a scattergram in Fig. 4. Based on these variables, 48 patients were categorized into three clusters with cluster analysis. Patient characteristics according to these clusters are shown in Table 2. The mean age at entry was similar among clusters. Frequencies of SCA, MSA-C, and CCA were significantly different among clusters (*p* = 0.007). The percent of patients with MSA-C was higher in Clusters 2 and 3. The annual atrophy of the corpus callosum (Adx) and cerebellum (Vdx) were significantly different among clusters (*p* = 0.001 for both; Table 2). The annual increases in the ICARS score were 2.9 ± 1.7 in Cluster 1, 4.8 ± 3.2 in Cluster 2, and 8.7 ± 6.1 in Cluster 3 (*p* = 0.014, Fig. 4).

**Table 2:** Patient characteristics by clusters

	Total (*n* = 48)	Cluster 1 (*n* = 12)	Cluster 2 (*n* = 22)	Cluster 3 (*n* = 14)	*p* value
Age at the first MRI (years)	60.5 ± 11.3	66.3 ± 8.5	60.4 ± 12.0	56.0 ± 10.6	0.088
Sex (Male/Female)	24/24	7/5	8/14	9/5	0.211
Diagnosis					
SCA, n (%)	21 (43.8)	6 (50.0)	11 (50)	4 (28.6)	0.007
SCA1	3	0	3	0	
SCA2	3	1	1	1	
SCA3	4	0	1	3	
SCA6	6	2	4	0	
SCA31	1	1	0	0	
SCA (unknown)	4	2	2	0	
MSA-C, n (%)	17 (35.4)	0 (0.0)	9 (40.9)	8 (57.1)	
CCA, n (%)	10 (20.8)	6 (50.0)	2 (9.1)	2 (14.3)	
ICARS score at the first visit	35.5 ± 16.5	39.1 ± 7.4	35.2 ± 22.2	30.0 ± 16.1	0.464
Initial cerebellar volume index (Vdx)	0.47 ± 0.09	0.45 ± 0.08	0.45 ± 0.10	0.50 ± 0.07	0.275
Annual atrophy volume in Vdx	−0.04 ± 0.02	0.02 ± 0.01*	−0.04 ± 0.01	−0.07 ± 0.02	0.001
Initial callosal area index (Adx)	3.38 ± 0.45	3.13 ± 0.39	3.53 ± 0.37^‡^	3.28 ± 0.46	0.011
Annual atrophy area in Adx	−0.11 ± 0.08	−0.04 ± 0.03^†^	−0.10 ± 0.05^†^	−0.19 ± 0.09^†^	0.001
Annual increase in ICARS score	4.4 ± 4.5	2.9 ± 1.7	4.8 ± 3.2	8.7 ± 6.1	0.014

*Vdx* cerebellar volume index, *ICARS* International Cooperative Ataxia Rating Scale

*Cluster 1 vs. Cluster 2, *p* < 0.05, Cluster 1 vs. Cluster 3, *p* < 0.05

^‡^Cluster 1 vs. Cluster 2, *p* < 0.05

^†^Cluster 1 vs. Cluster 2, *p* < 0.05, Cluster 1 vs. Cluster 3, *p* < 0.05, Cluster 2 vs. Cluster 3, *p* < 0.05 (post-hoc Dunnett’s test)

## Discussion

Designing clinical trials to treat degenerative cerebellar ataxia has been challenging because differential diagnosis is usually difficult in the early stage when treatment would be most effective, and the rate of progression may vary, even in patients with the same clinical subtype or the same genotype [7]. Our study demonstrated that symptom progression significantly differed by subtype of cerebellar ataxia, especially both the annual cerebellar/corpus callosum atrophy rating and increase in ICARS score were highest in MSA-C patients. However, the standard deviations for these values were relatively large, even in the same subtype. Schmitz-Hubsch et al. reported that 250 patients per group were required to detect a 50% reduction in disease progression as evaluated with the Scale for Assessment and Rating of Ataxia (SARA) in a two-arm trial with the outcome measured within 1 year, implying that the sample size estimate was still large, even in patients with genetically proven etiology [30]. Detailed characterization of phenotypes of degenerative cerebellar ataxia is essential for identification of responder populations to allow medical intervention. Our study is the first to explore different subtypes of disease progression through cluster analysis in an attempt to overcome this sample size problem.

MRI-based cerebellar volume measurement has been proposed as an imaging biomarker to predict differences in progression rates in these diseases [1, 19–21]. The relationship between MRI-based cerebellar volume measurements and the SARA score has been extensively studied in several types of SCA [1]. However, MRI-based cerebellar volume measurements have never been established as a stratification tool to categorize disease progression. Recently, we demonstrated that the annual atrophy rate of the cerebellar volume and the annual progression of the ICARS score are significantly different among subtypes of cerebellar degeneration, but categorizing individual symptom progression only with the atrophy rate of the cerebellum is not feasible [22, 23]. Our current study demonstrated that cluster analysis based on the annual atrophy rates of the corpus callosum and cerebellum successfully categorized the difference in disease progression in a broad spectrum of degenerative cerebellar ataxias. This simple MRI method may be useful as a stratification tool during the run-in period of clinical trials.

We adopted the ICARS scoring system, a validated, 100-point ordinal scale (higher scores indicate greater ataxia). ICARS scoring is sensitive across a range of ataxia severities, from very mild to severe, and the inter-rater reliability is very high [31]. However, ceiling effects have been reported in more advanced stages of disability with an ICARS sum score above 60 [32]. As shown in Fig. 3, the ICARS score was similarly increased in all patients, and an initial ICARS score above 60 was found only in two patients. A strength of our study was the long follow-up period. Because of the gradual progression of this disease, cluster analysis may not sensitive enough to detect the difference in progression speed in a short period. Our study suggests that follow-up of around 18 months may be feasible for stratifying patients with different progression speeds.

Our study has some limitations. First, our study was a retrospective analysis using longitudinal follow-up data. Brain MRI and ICARS scores were assessed around the same time, but the study period was not established beforehand, and no prospective data were collected. Therefore, the follow-up periods and number of examinations differed among patients. Second, we did not use the initial diagnosis but the final diagnosis, and the age of onset was based on the medical history given by each patient. Third, we retrospectively analyzed 2D MRI data with 4-mm slice thickness obtained by routine examination. Nowadays, high-resolution 3D MR imaging can be easily performed, and several sophisticated MRI methods have been developed for the measurement of cerebellar volume [33–35]. In designing clinical trials, much more sensitive MRI methods than used in this study could be selected to detect changes in atrophy.

## Conclusions

Our study is the first to demonstrate the utility of cluster analysis based on the atrophy rates of the corpus callosum and cerebellum to categorize gross neurological deterioration as evaluated by ICARS. Further studies are warranted to explore whether these simple MRI methods could be used for random allocation of patients with a broad spectrum of degenerative cerebellar ataxias in clinical trials, for example, by setting up a run-in phase.

## Abbreviations

AC-PC line: Anterior commissure-posterior commissure line
AP: Antero-posterior
CCA: Cortical cerebellar atrophy
ICARS: International cooperative ataxia rating scale
MRI: Magnetic resonance imaging
MSA: Multiple system atrophy
MSA-C: Multiple system atrophy, especially the cerebellar type
SARA: Scale for the assessment and rating of ataxia
SCA: Spinocerebellar ataxia
